# Psychological treatment of depressive symptoms in Chinese elderly inpatients with significant medical comorbidity: A meta-analysis

**DOI:** 10.1186/1471-244X-11-92

**Published:** 2011-05-20

**Authors:** Bibing Dai, Juan Li, Pim Cuijpers

**Affiliations:** 1 Center for Ageing Psychology, Key Laboratory of Mental Health, Institute of Psychology, Chinese Academy of Sciences, Beijing, China; 2 Graduate School, Chinese Academy of Sciences, Beijing, China; 3 Department of Clinical Psychology and the EMGO Institute for Health and Care Research, VU University Amsterdam, The Netherlands

## Abstract

**Background:**

As it is uncertain whether psychological treatments for depressive symptoms are effective in elderly inpatients with significant medical comorbidity, we aimed to assess the treatment effectiveness not only on depressive symptoms but also on somatic symptoms in these inpatients.

**Methods:**

We performed a meta-analysis of randomized controlled studies assessing the effects of psychological treatments in Chinese older inpatients with significant medical comorbidity based upon extensive searches of the most comprehensive computerized Chinese academic database.

**Results:**

The overall effect size for depressive symptoms of twelve studies which compared psychological treatments with a care-as-usual control group was *d *= 0.80 (95% Confidence Intervals (CI) = 0.60-0.99; *p *< 0.001). The relative risk of psychological intervention of being effective or not, compared to control condition, was 1.52 (95% CI = 1.25-1.85; *p *< 0.001).

**Conclusions:**

We conclude that psychological treatments of depressive symptoms are effective for Chinese elderly inpatients with significant medical comorbidity which should receive more attention in medical settings.

## Background

Depression is a common mood disorder that can lead to considerable suffering by patients and their relatives, physical, cognitive and social dysfunction, a significantly increased mortality rate and a massive economic burden [1-3]. It is well acknowledged that the prevalence of depression in late life is high with major depression ranging from 1% to 5%, and clinically significant depressive symptoms varying between 8.3% and 15% [4,5], while it is even higher in older inpatients with significant medical comorbidity [6]. The prevalence of major depression among older inpatients with significant medical comorbidity ranges from 10% to 25%, and for clinically significant depressive symptoms from 23% to 28% [7,8]. In old age, depression is particularly prevalent in patients with cardiovascular disease [9], stroke [10], Parkinson's disease [11], diabetes [12], and Alzheimer disease [13]. It is also widely acknowledged that significant medical comorbidity may interact with depression [9,14]. On the one hand, depression in elderly inpatients often amplifies their physical symptoms, impairs their ability to adhere to medication, and causes higher levels of morbidity and disability [7,15]. On the other hand, there is considerable evidence that significant medical comorbidity can produce a depressive reaction [14,16].

In a similar way to many Western countries, depression also results in a great deal of negative effects in China. Specifically, the cost of treatment for depression in China was estimated to be approximately 1.0% of the total national health care costs in 2002 (US$ 814 million out of US$ 82,385 million) [17]. In consideration of the rapid growth of the elderly population and the high prevalence of depression in older adults that the rates of major depression and clinically significant depressive symptoms are 3.82% and 8.8%, respectively in China [18,19], the cost used for treatment of late life depression could be enormous, which may include the health care cost used for elderly inpatients with significant medical comorbidity.

Several meta-analyses have shown that psychological therapies are effective in treating depression in the general elderly population [20,21], but it is not yet clear whether psychological treatments are effective in older depressed inpatients with significant medical comorbidity. Because older depressed inpatients with serious medical comorbidity may show lower response to psychological treatment for depression, and their depressive symptoms may be due to physical changes [22], all these make it more difficult to treat their depression. Furthermore, although there is substantial evidence that significant medical comorbidity may interact with depression, it is not known whether psychological treatments for depression also have positive effects on medical comorbidity, which has not been explored by meta-analysis yet.

Taking into consideration the fact that depression in elderly inpatients with significant medical comorbidity could cause a great deal of negative effects, a lot of studies using psychotherapies originated in Western countries (such as cognitive behavior therapy and non-directive supportive therapy) have occurred in China recently. However these studies and the journals they were published in are local, they are usually not easily accessed by Western researchers. As a result, it is not well known whether the psychotherapies widely used in Western countries are also efficacious for this special population in this field. However, it is important from a clinical point of view, because this would indicate that these psychotherapies have a clinical meaning in non-Western societies as well. Meanwhile, it is also interesting from a scientific point of view, because if these psychotherapies are effective this implies that they are not only linked with Western customs and habits, but can have a broader meaning.

Therefore, we decided to conduct a meta-analysis to focus exclusively on the effectiveness of psychological treatments for Chinese elderly inpatients with significant medical comorbidity. First, we examined whether psychological treatment of depression was effective in reducing the level of depression. Second, we examined whether psychological treatment of depression was effective in mitigating the somatic symptoms.

## Method

### Selection of studies

Studies were selected through a systematic search of a computerized database of the literature (China National Knowledge Infrastructure; CNKI) which is the most comprehensive Chinese academic database. We used 'elderly or old age or aged', 'depression or depressive symptoms' and 'psychological treatment or psychological intervention or psychotherapy' as search themes in the titles, keywords or article abstracts. The search was conducted from 1964 to the end of 2008.

### Inclusion and exclusion criteria

We included all studies in which: (1) the subjects were aged 60 or older; (2) the subjects were inpatients with significant medical comorbidity; (3) the subjects had a depressive disorder according to clinical diagnosis as described in DSM or Clinical diagnosis according to the Chinese Classification of Mental Disorders (CCMD), or the mean depression scores (measured with Self-rating Depression Scale or Symptom Checklist 90-Depression) of both experimental and control groups were significantly higher than the norms of Chinese elderly or above the cutoff value widely accepted by Chinese practitioners in this field; (4) the subjects from both experimental and control groups did not use antidepressant medication; (5) a psychological treatment was compared to a control group; (6) a randomized controlled trial was conducted. We excluded studies on subjects below 60 years of age. Also excluded were studies in which the depressed inpatients did not have medical comorbidity; studies in which the effects of the psychological treatment could not be distinguished from the total intervention and studies in which insufficient data were available to calculate effect sizes. Eligibility judgment and data extraction were carried out independently by two researchers. All disparities between them were resolved by consensus.

### Quality assessment

Although many scales are available to assess the validity and quality of trials, none can provide an adequately reliable assessment. Therefore, we selected a number of basic criteria for assessing the validity of the studies, as suggested by the Cochrane Handbook [23], which are frequently used in meta-analysis of psychological treatments for depression [20]. There are four basic criteria in the Cochrane Handbook: allocation to conditions by an independent (third) party; blinding of assessors of outcomes; adequacy of random allocation concealment to respondents and completeness of follow-up data. Because it was impossible for psychological treatment studies to conceal random allocation to subjects adequately, we did not use the third basic criteria in the present study. The assessment was conducted by two independent reviewers and all disparities between them were resolved by consensus.

### Meta-analyses

We computed the effect size *d *for each study by subtracting the average post-test score of the control group (M_c_) from that of the experimental group (M_e_) and dividing the result by the pooled standard deviations of the experimental and control groups (SD_ec_). or example, an effect size of 1.0 would indicate a relatively stronger improvement in treatment by one standard deviation larger than the mean of the control group. To interpret the practical significance of the results, we used Cohen's criteria [24]. Effect sizes of 0.80 are regarded as large, while effect sizes of 0.50 are moderate, and effect sizes of 0.2 are small. When means and standard deviations of the studies were not provided, we used other statistics (χ^2 ^value and *p *value) to compute effect sizes with the help of Comprehensive Meta-Analysis (CMA; version 2.2.046). Pooled effect sizes and 95% confidence intervals (CI) were calculated according to the procedures implemented in CMA.

As considerable heterogeneity was found among these studies, we decided to calculate mean effect sizes with the random effects model. As indicators of heterogeneity, we computed the *Q *statistic and *I^2^*. *I^2 ^*denotes the variance among studies as a proportion of the total variance. The larger the value of *I^2 ^*is, the greater the heterogeneity. An *I^2 ^*of 0% shows no observed heterogeneity, while 25% shows low, 50% moderate, and 75% high levels of heterogeneity [25]. We also computed the *Q *statistic and reported whether it was significant or not. If the *p *value is above 0.05, it indicates that there is no significant heterogeneity and that the total variance results from the variance within studies rather than from the variance between studies.

Finally, publication bias of the included studies was examined by visual inspection of the funnel plot on the primary outcome measure, and by Duval and Tweedie's trim and fill procedure, which provides an estimate of the effect sizes after publication bias has been considered (as implemented in CMA).

## Results

### Study selection

**Figure 1:**
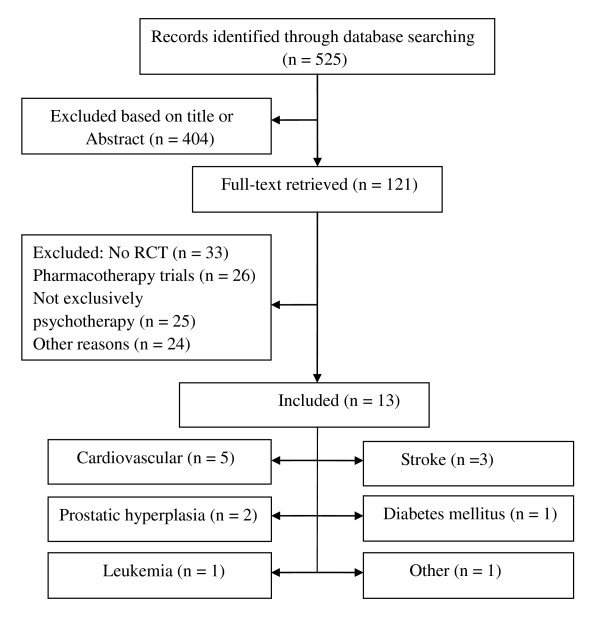
**Selection of studies for the review**.

A flowchart describing the inclusion process is presented in Figure 1. We identified a total of 525 possibly eligible papers. The titles and abstracts of these 525 papers were studied and 121 were selected for further examination. Based on the full text of these 121 papers we finally selected 13 studies for the present meta-analysis [26-38]. The most important reasons for exclusion were: not being a randomized controlled trial (n = 33), the patients used antidepressant medications (n = 26), and the effects of the psychological treatment could not be distinguished from the total intervention (n = 25). Other reasons included that the patients were not inpatients or without significant medical comorbidity.

### Description of studies

Of the thirteen included studies five were conducted among inpatients with cardiovascular disease, three among inpatients with stroke, two among inpatients with prostatic hyperplasia, one among inpatients with diabetes mellitus, one among inpatients with leukemia, and one among inpatients with unspecified medical comorbidity (Figure 1). In all of these cases, the significant medical comorbidities were confirmed by the physicians on the basis of physical examinations. All the studies compared psychotherapy to a care-as-usual group. All psychological treatments in the experimental groups consisted of integrative psychotherapy, of which non-directive supportive therapy (SUP) and cognitive behavior therapy (CBT) were the dominant components. There were five studies in which the psychotherapy was delivered in individual face-to-face format and eight studies in which the psychotherapy was delivered in mixed format incorporating both individualized and group treatments (Table 1). Finally, the depression score at post-test was assessed by clinicians in two studies, while that of the other eleven studies was assessed by self-report questionnaires (e.g., Self-rating Depression Scale).

**Table 1:** Characteristics of included studies

*Author & Year*	*Comorbidity*	*Age*	*Definition of depression*	*Conditions*	*N*	*N_se_*	*Frm*	*Depression measures*	*Clinical index*
Du, 2007	Atrial fibrillation	60-80; M = 66.0	Other definition (M_SDS _= 55.25)	1.CBT + SUP + Health education + Relaxation+ Music therapy	30	10	Ind	SDS	The number different curative effects
				2. Care-as-usual	30				
Hu & Gui, 2007	Leukemia	60-82	Other definition (SDS > 40; M_SDS _= 53.77)	1. SUP + Teleotherapeutics	25	-	Ind	SDS	-
				2. Care-as-usual	25				
Kong & Li, 2004	Stroke	60-82; M = 62.7	Clinical diagnosis (DSM-3-R; HRSD ≥ 13; M_HRSD _= 20.53)	1. CBT+SUP	34	-	Mixed	HRSD	The number different curative effects
				2. Care-as-usual	30				
Li, 2007	Somatic disease	M = 74	Other definition (SDS > 40; M_SDS _= 62.22)	1. CT + SUP	27	-	Ind	SDS	-
	(-)			2. Care-as-usual	25				
Li *et al*., 2007	Prostatic hyperplasia	60-76	Other definition (M_SDS _= 54.70)	1. SUP + Relaxation + Health education + Music therapy	40	-	Mixed	SDS	The number of spastic bladder; The number of using anodyne
				2. Care-as-usual	40				
Liu et al., 2000	DM	60-79; M = 63.1	Other definition (M_SCL-90-D _= 1.73)	1.CT+ SUP +Health education + Relaxation + Music therapy	32	-	Mixd	SCL-90-D	The number of different curative effects
				2. Care-as-usual	32				
Meng, 2007	CHD	63-83; M = 75.2	Other definition (M_SDS _= 56.23)	1. SUP + Relaxation therapy +Music therapy	32	-	Mixed	SDS	SAQ; The number of different curative effects
				2. Care-as-usual	32				
Qu, 2002	CHF	≥ 65; M = 66.5	Other definition (M_SDS _= 55.32)	1. CBT + SUP	28	16	Ind	SDS	The number of different curative 24 effects
				2. Care-as-usual	28				
Shu & Dong, 2008	CHD	65-84	Other definition (M_SDS _= 53.15)	1.CBT+ SUP + Relaxation + Health education +Music therapy	20	8	Mixed	SDS	-
				2. Care-as-usual	20				
Tang *et al*., 2008	Stroke	61-85; M = 65.5	Clinical diagnosis (CCMD-3, HRSD ≥ 17; M_HRSD _= 22.31)	1. CT + SUP	29	-	Mixed	HRSD	BI; FMA; NFA
				2. Care-as-usual	29				
Xie & Jiang, 2005	BPH	60-78; M = 67.3	Other definition (M_SCL-90-D _= 1.68)	1. BT + SUP + Health education	35	-	Mixed	SDS	SF-36
				2. Care-as-usual	30				
Zhou et al., 2008	Hypertension	65-80; M = 72.7	Other definition (SDS ≥ 50; M_SDS _= 53.7)	1.SUP + CT + Relaxation therapy	50	16	Mixed	SDS	SBP, DBP
				2. Care-as-usual		50			
Zhu *et al*., 2007	Stroke	60-82; M = 66.1	Other definition (M_SCL-90-D _= 2.13)	1. CT + SUP + Health education	31	18	Ind	SCL-90-D	-
				2. Care-as-usual	31				

Abbreviations: BI-Barthel Index; BPH, benign prostatic hypertrophy; CCMD, Clinical diagnosis according to the Chinese Classification of Mental Disorders; CBT, cognitive behavior therapy; CHF, chronic heart failure; CHD, coronary heart disease; Clinical index, The measures were used for assessment of effect of psychological interventions on significant medical comorbidity; CT, cognitive therapy; DBP, Diastolic blood pressure; DM, diabetes mellitus; Frm, format; HRSD, Hamilton Depression Scale; Ind, individual format; Mixed, format incorporating both individualized and group treatment; NFA, Neurological function assessment; Nse, number of sessions; SAQ, Seattle Angina Questionnaire; SBP, Systolic blood pressure; SCL-90-D, Symptom Checklist 90-Depression; SDS, Self-rating Depression Scale; SF-36, 36-item short-form healthy survey; SUP, Non-directive supportive therapy; the number of different curative effects, the number of participants whose curative effects of significant medical comorbidity are effective (very effective, effective) or not effective (barely effective, not effective); -, no report.

### Quality assessment

The quality of the studies was not optimal. None of the thirteen studies reported whether allocation to conditions was conducted by an independent party. In eleven studies self-rating scales were used (and so blinding of assessors was not relevant), while two studies did not provide information whether assessors were blinded. Finally, the absence of drop-out in all included studies indicated a completeness of follow-up data.

### Effects of psychological treatments on depression compared to care-as-usual

**Figure 2:**
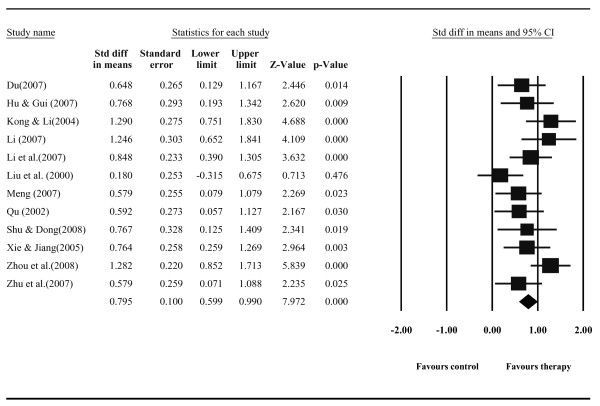
**Post-treatment effect sizes of psychological treatment for depressive symptoms in inpatients with significant medical comorbidity compared to care-as-usual**.

Thirteen studies reported post-test effects of psychological treatment on depressive symptoms compared with care-as-usual control group, with a total of 816 respondents (413 people in the experimental condition, and 403 people in the control condition). The random effects model showed an overall effect size of *d *= 0.96 (95% CI = 0.63-1.28; *p <*0.001). However, the homogeneity analysis of the effect sizes (*Q *= 58.52, *p *< 0.001; *I^2 ^*= 79.49%) showed that there was considerable heterogeneity. After removal of one outlier whose effect size fell out of 3 SD from the mean effect size [39], the remaining studies included a total of 758 respondents with 384 people in the experimental condition, and 374 people in the control condition. The random effects model analysis showed an overall effect size of *d *= 0.80 (95% CI = 0.60-0.99; *p *< 0.001) and low to moderate heterogeneity (*Q *= 18.66, *n.s*.; *I^2 ^*= 41.04%). The effect sizes and 95% confidence intervals of the individual contrast groups are plotted in Figure 2. These analyses indicate that psychological treatments have large effects on depressive symptoms in elderly inpatients with significant medical comorbidity.

### Effects of psychological treatments on somatic outcomes

**Figure 3:**
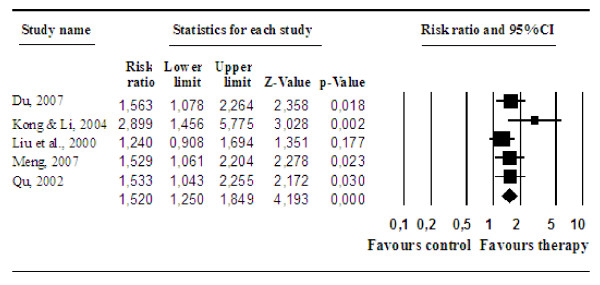
**Post-treatment effects of psychological treatment for depression in inpatients with significant medical comorbidity on the somatic treatment, compared to care-as-usual: Relative risk**.

There were nine studies in which the treatment effects on medical comorbidity were reported (Table 1). After excluding four studies either using self-reports which may be affected by patients' bias or in which it was difficult to characterize whether the treatment of medical comorbidity was successful or not, five studies with 305 respondents (156 people in the experimental groups, and 149 people in the control groups) were included in the analysis of the effects of psychological treatments on somatic outcomes. In these five studies, it was indicated whether the treatment was very effective, effective, barely effective or not effective according to clinically objective indicators (e.g. electrocardiograph examination or blood glucose levels). We dichotomized these outcomes was effective (very effective, effective) or not effective (barely effective, not effective), and calculated the relative risk (RR) of the intervention of being effective or not, compared to the control condition. The pooled RRs were also calculated with the random effects model. The results showed a RR of 1.52 (95% CI = 1.25-1.85; *p *< 0.001), with low heterogeneity (*Q *= 5.01, *n.s*.; *I^2 ^*= 20.17%). The effect sizes and 95% confidence intervals of the individual contrast groups are plotted in Figure 3. The result indicates that psychological treatments have moderate effects on medical comorbidity of depressed elderly inpatients.

### Publication bias

Visual inspection of the funnel plot indicated some publication bias. Duvall and Tweedie trim and fill procedure resulted in an effect size of *d *= 0.88 (95% CI = 0.68-1.09; number of imputed studies: 2), which suggesting that the results of the study were not significantly altered after adjusting for the publication bias.

## Discussion

In the present study, we analyzed the effects of psychological treatments on depressed Chinese elderly inpatients with significant medical comorbidity. Psychological treatments showed large effects on their depressive symptoms, which is similar to the findings of psychological treatments for depression in older adults in the general population [20,40]. Some studies found that older depressed adults with significant medical comorbidity may show lower response to psychological treatments for depression [22], while other revealed that psychological treatments for late-life depression are effective among the terminally ill [41]. The present result may be due to the following reasons. First, from the perspective of life-span development, older adults still have the ability to acquire new knowledge and skills and to use them in their daily life [42]. Second, given that there were high levels of comorbidity in the samples, which increased the complexity of treatment, the integrative psychotherapy focusing on different types of behaviors, problems, or symptoms may be advantageous [43,44]. All the psychological treatments in the present study comprise integrative psychotherapy, which may increase the treatment effects. Third, compared to outpatient samples who may have higher drop-out rates because of transportation and competing demands [21], all of the samples in our study were inpatients who had adequate time and appropriate locations to receive psychological treatments, thus with a reduced risk for treatment drop-out. Recent studies emphasized the issues of compliance and dropout in treatment research on older people, and claimed that the drop-out rate served as an important indicator of therapeutic effectiveness [45]. Therefore, the large treatment effect in the present study may be due to the absence of drop-out. Fourth, the psychological treatments were executed by doctors who are highly respected by patients in Chinese culture, which may have improved not only compliance but also motivation for receiving treatment in patients. In addition, it is very well possible that the inpatients were concerned that it would reduce the quality of care they receive from their doctors if they refused to participate in the interventions initiated by their doctors. This is also reflected by absence of drop-out in all of the studies. Fifth, having a care-as-usual control group rather than an active control group (such as other psychotherapy or pharmacotherapy) may have increased the effect sizes.

Since depression may influence treatment for significant medical comorbidity in patients, the psychological treatment of depression might improve the functional health of patients, contributing to an improvement in their significant medical comorbidity as well. This has been rarely examined in previous studies [7,46]. Furthermore, there is no meta-analysis to test this issue yet. In our present study, we found that psychological treatments have moderate effects on medical comorbidity among Chinese elderly inpatients. For example, psychotherapies could improve somatic function, increase quality of life and hasten recovery.

The present study has several limitations. First, a relatively small number of studies were used in this meta-analysis, which means the results should be interpreted with caution. The low number of studies also limits the possibility of conducting subgroup analyses to identify some potential important moderators such as the categories of significant medical comorbidity, the treatment formats or treatment intensity that may also affect the effect sizes. Second, we found that the quality of the included studies in the present study was not optimal. For example, many studies did not report whether assignment to conditions was executed by an independent person, or whether blinding of assessors was conducted. Third, because follow-up results after post-test were not reported, we do not know whether there are long term effects. Fourth, the psychological treatments of all studies were integrative, so we could not compare the effects of different psychotherapies in this special population. Fifth, all studies were conducted in China, so whether the present results could be extended to Western populations needs a more comprehensive meta-analysis including studies conducted in Western countries.

Despite these limitations, we firmly conclude that psychological treatments are efficacious for Chinese elderly inpatients with significant medical comorbidity. Though the point was concluded based upon Chinese samples, it may still have important implications. First, there is a high comorbidity rate in older adults' depression and physical diseases. Second, a large number of inpatients with significant medical comorbidity who suffer depression go undetected and untreated [7]. And third, older adults prefer receiving psychotherapy to taking antidepressant medication due to the adverse effects of antidepressants [47]. Therefore, general practitioners should pay more attention to psychological treatments of the depressive symptoms in older inpatients with significant medical comorbidity in medical settings, as psychological treatments are not only effective for reducing depressive symptoms, but also efficacious for alleviating somatic symptoms. Another important and helpful advice from the present research is that the therapists should also pay more attention to improving the patients' motivation for psychological treatments in order to reduce the drop-out rate in this population. In addition, this study also suggested that the psychotherapies widely used in Western countries are also efficacious in Eastern culture context.

## Conclusions

We conclude that psychological treatments of depressive symptoms could mitigate both depressive symptoms and somatic symptoms in Chinese elderly inpatients with significant medical comorbidity.

## Competing interests

The authors declare that they have no competing interests.

## Authors' contributions

BBD, JL and PC together initiated the idea for the meta-analysis. BBD collected the data, conducted the analyses, and wrote the paper. JL supervised the data collection, statistical analysis and paper writing. PC helped with the analyses and reviewed the texts critically. All authors have read and approved the final manuscript.

## Pre-publication history

The pre-publication history for this paper can be accessed here:

http://www.biomedcentral.com/1471-244X/11/92/prepub
